# Qualitative Analysis of a Remote Monitoring Intervention for Managing Heart Failure

**DOI:** 10.21203/rs.3.rs-2206783/v1

**Published:** 2023-01-20

**Authors:** Tamar Klaiman, L.G. Ianotte, Michael Josephs, Louise B. Russell, Laurie Norton, Shivan Mehta, Andrea Troxel, Jingsan Zhu, Kevin Volpp, David Asch

**Affiliations:** University of Pennsylvania Perelman School of Medicine; Lake Erie College of Osteopathic Medicine; University of Pennsylvania Perelman School of Medicine; Rutgers University New Brunswick; University of Pennsylvania Perelman School of Medicine; University of Pennsylvania Perelman School of Medicine; New York University Medical Center: NYU Langone Health; University of Pennsylvania Perelman School of Medicine; University of Pennsylvania Perelman School of Medicine; University of Pennsylvania Perelman School of Medicine

**Keywords:** Heart failure, remote monitoring, qualitative interviews

## Abstract

**Background:**

Heart failure (HF) is one of the most common reasons for hospital admission and is a major cause of morbidity, mortality, and increasing health care costs. The EMPOWER study was a randomized trial that used remote monitoring technology to track patients’ weight and diuretic adherence and a state-of-the-art approach derived from behavioral economics to motivate adherence to the reverse monitoring technology.

**Objective:**

The goal was to explore patient and clinician perceptions of the program and its impact on health outcomes and better understand why some patients/clinicians did better/worse than others in response to the intervention.

**Approach:**

This was a retrospective qualitative study to understand the trial’s processes, reflecting on successes and areas for improvement for future iterations of behavioral economic interventions.

**Key Results:**

Many patients felt supported, and they appreciated the intervention. Many also appreciated the lottery intervention, and while it was not an incentive for enrolling for many respondents, it may have increased adherence during the study. Clinicians felt that the intervention integrated well into their workflow, but the number of alerts was burdensome. Additionally, responses to alerts varied considerably by provider, perhaps because there are no professional guidelines for alerts unaccompanied by severe symptoms.

**Conclusion:**

Those interviews offer insights into the potential reasons for the study’s null result and opportunities for improvements in the future.

**Trial Registration:**

ClinicalTrials.gov Identifier: NCT02708654

## Background

Heart failure (HF) is one of the most common reasons for hospital admission and is a major cause of morbidity, mortality, and increasing health care costs.^1, 2^ The EMPOWER study aimed to reduce readmissions or mortality in this population.^3^ This randomized trial assessed a comprehensive intervention that combined remote monitoring technology to track patients’ weight and diuretic adherence, alerting clinicians to significant changes via the electronic medical record (EMR), with a state-of-the-art approach derived from behavioral economics to motivate adherence to the remote monitoring technology.

The study equally randomized 552 patients to a control or an intervention arm; control patients were not contacted again after enrollment. The trial followed patients for a year and found no effect of this comprehensive intervention on readmissions or mortality.^3^

Before those results were known, a qualitative interview study was designed and implemented to understand the trial’s processes, reflecting on successes and areas for improvement for future iterations of behavioral economic interventions. The goal was to explore patient and clinician perceptions of the intervention and its impact on health outcomes^3^ and better understand why some patients/clinicians did better/worse than others in response to the intervention. The interviews offer insights into the potential reasons for the study’s null result and opportunities for future improvements.

## Methods

The EMPOWER study was a 2-arm randomized clinical trial that took place May 2016 – April 2020; details of its design have been described elsewhere. ^3, 4^ Patients were recruited following discharge from one of three Philadelphia hospitals in the University of Pennsylvania Health System (UPHS), as long as their care was to be later managed by a UPHS clinician. All participants randomized to the intervention arm received (1) a digital scale, (2) an electronic pill bottle (LLC Technologies) that chirped to remind patients to take their daily diuretic medication, and (3) daily reminders and lottery incentives with an approximately 1 in 5 chance of a $5 payout and a 1 in 100 chance of a $50 payout based on both medication adherence and weight measurement from the previous day. The trial’s primary outcome was time to readmission for any cause or death.

Each participant’s weight was monitored daily, and weight increases were automatically compared against an increase of three pounds in 24 hours or five pounds over three days. Pill bottles were also monitored daily to see if they were opened. If a weight change exceeded one of these thresholds, or a pill bottle was not opened the previous day, the patient was contacted by study staff to verify the weight and medication consumption and answer a symptom questionnaire. This information was then sent as an abnormal result into the electronic medical record (EMR) and routed to the patient’s managing clinician. Additionally, if a patient reported worsening shortness of breath or chest pain, the study team called their clinician. Patients not reached by the study team after three calls also had an alert sent to their EMR indicating their weight change and/or medication adherence with a note that they were unreachable. All verified weights were also sent into a flowsheet in the participant’s EMR on a weekly basis. We tracked whether each alert was acknowledged—that someone from the patient’s care team opened the abnormal result. We also tracked how clinicians *responded* to each alert: MyPennMedicine encounters (patient messaging portal), telephone encounters, lab orders, changes to diuretics, emergency department or clinic referrals, clinician to clinician contact (discussing patient care with another team member). We evaluated clinician responses to all adherence alerts triggered in the EMR.

## Patients

To be eligible to participate in the trial, participants had to be prescribed a daily diuretic and plan to have their HF monitored by a Penn Medicine clinician (a primary care clinician, cardiologist, or nurse practitioner). Exclusion criteria included receipt of heart transplant, listed or under evaluation for heart transplant, receipt of ventricular assist device, listed or under evaluation for ventricular assist device, end-stage renal disease, a glomerular filtration rate < 25 mL/min, hemodialysis, inotrope dependence, palliative care, hospice care, or participation in other telemonitoring interventions. In addition, participants were ineligible if they had a history of uncontrolled cognitive or psychiatric conditions that would affect study participation.^4^

For this qualitative study, patients who had graduated 3 months prior to their interview being scheduled were sampled so they could reflect on their behavior after the study. A 2 × 2 factorial design was implemented based on whether their intervention adherence was high (> 95% for both devices) or low (≤ 70% for both devices) and whether they were readmitted or not. These cut points represented the 25th and 75th percentile of adherence based on all patients. Adherence was measured beginning on the date the patient enrolled in the study. We called each identified patient with the goal of interviewing 10 respondents per quadrant. See Table 1 for recruitment demographics. Each patient received up to three recruitment telephone calls. The final sample constitutes a convenience sample of patients who responded to a request for participation. Low adherents were particularly challenging to recruit.

Research coordinators (LGI and MJ) called identified individuals to invite them to participate. Each individual was asked for verbal consent to a 45–60-minute interview. Interviews were semi-structured, and participants received a $50 payment for participation. All interviews were tape recorded and professionally transcribed for analysis.

The Senior Qualitative Research Scientist (TK) assisted with initial coding and the development of a codebook. Two research coordinators (LGI and MJ) coded each interview in NVIVO 12. Codes were compared at the completion of each set of coding, and any discrepancies in coding were discussed and adjudicated by the Research Scientist (TK). The study was approved by the Institutional Review Board of the University of Pennsylvania (PROTOCOL #824816).

## Clinicians

We identified practices with patients who were active in the EMPOWER intervention in 2019, the final full year of the intervention. To ensure clinicians were recently engaged with the intervention, we recruited those in the top half by number of patient alerts received during 2019. We conducted brief (15–30 minute) interviews with clinicians who agreed to participate. Only clinicians whose patients had already graduated from the study were included to reduce bias in physician behavior from interviews. Physicians, nurse practitioners, and nurses were eligible to participate. Participants received a $25 Amazon gift card for participating in the qualitative interviews. Data were analyzed using the same process as patient interviews.

## Results

### Patients

Forty-three patients of the 62 recruited (70%) agreed to participate. Information about participants is noted in Table 2, selected quotes from the interviews in Table 3.

#### Health Perception

As a condition of intervention enrollment, all participants had been hospitalized for HF-related issues. Most respondents had co-morbidities including diabetes, hypertension, atrial fibrillation, stroke, and peripheral artery disease. Most participants perceived improved health status because of the intervention. All respondents felt that the intervention helped them manage their heart failure and improved or helped to maintain their routines, *“…so I knew if I had to eat more salad.”* Post intervention perceptions of health status varied by patient, but not by adherence level. About a third of patients missed the intervention and believed it helped them to improve their health.

#### Disease Monitoring and Routine

Participants generally liked the monitoring devices and experienced few issues during set-up and use. All respondents were able to get their medication without problems. Most participants stated that they appreciated the daily reminders to take medication, *“The pill bottle… put structure in my life.”* Some participants did not weigh themselves regularly prior to enrolling in the intervention; some participants did not have a scale, while others only weighed themselves when they had symptoms or remembered to do so. All participants stated that the intervention helped them to remember to weigh themselves and take their medication more regularly. Most integrated these activities into their daily routines, and some felt that the pill bottle reminder helped them remember to weigh themselves, *“It actually reminded me … I would step on the scale.”* Almost all highly adherent patients continued to weigh themselves daily after the intervention ended, *“I still weigh myself every day,”* while all low adherence patients reported weighing themselves less after the intervention ended.

#### Effectiveness

All patients stated that participation in the intervention helped them increase their awareness about their heart failure, although some did not understand the connection between their weight and heart failure. One respondent stated *“And I’m not sure how just taking the medicine and getting weighed told you anything about the heart...”*

Although the trial showed no statistically significant effect of the intervention on readmissions,^3^ almost all of the high adherence, no readmission patients felt that intervention helped them stay out of the hospital, *“I think the EMPOWER program helps you not go to the hospital,”* while a majority of the low adherence, no readmission patients felt that way. Almost all of the high adherence, readmission patients felt that the intervention kept them out of the hospital more than would have been the case if they had not been in the program, while low adherence patients who were readmitted did not mention the program or its impact on hospitalizations.

#### Lottery

All respondents cited their desire to better manage their heart failure as their motivation for enrolling. No patients cited the lottery as their main reason for joining the intervention, but many stated that it factored into their decision, *“I was still gonna be in it. But it just made it a little better.”* Others said that, while the lottery did not affect their decision to enroll, they liked and appreciated it. Most patients said that they found the lottery to be motivating/encouraging to stick with weighing themselves daily and taking their medications as prescribed, *“it was more a case of making sure I did what I was supposed to do.”* Two patients also cited an altruistic desire to help other heart failure patients as a motivation for joining the EMPOWER research study.

#### Alerts

**Figure 1:**
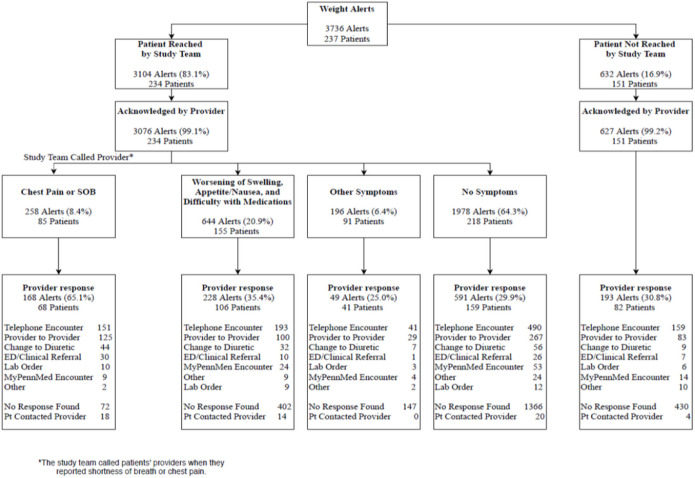
Weight Alerts

Over 3,500 alerts were triggered among 237 intervention patients during the trial (Fig. 1). Among patients who experienced severe symptoms associated with exacerbated heart failure such as chest pain or shortness of breath (8.4% of alerts), the majority (65.1%) received outreach from the clinician’s office; however, among those with other heart failure symptoms such as edema or nausea (20.9% of alerts), only 35.4% received a response from clinicians. Patients with symptoms not associated with worsening heart failure or no symptoms were the least likely to receive any response from clinicians’ offices. Over 60% of alerts were triggered for patients who had no symptoms; of those, fewer than 30% of alerts received a response from a clinician. Telephone encounters were the primary method of interaction between clinicians and patients after alerts were triggered. The most common intervention was a change to diuretics; however, that only occurred in a small number of incidents (12%).

### Clinicians

Thirty-four clinicians were recruited, and 16 (47%) participated in interviews. Respondents included 9 physicians, 5 nurses, and 2 nurse practitioners. We conducted 15–30-minute semi-structured interviews via telephone.

#### Workflow

All respondents felt that managing alerts integrated easily into their existing clinic workflow. Clinicians appreciated that the alerts were in the EMR and could be managed utilizing existing processes. Each patient had a weight flowsheet in the EMR that clinicians could access to review trends over time. Clinicians reviewed alerts, previous encounters, and flowsheets to see if they had recently interacted with the patient before reaching out. If there were no symptoms, some clinicians would make a note to check in on the patient in a week or two. Other clinicians could not really recall reviewing flowsheets of patient weights. A few clinicians felt that the data were hard to analyze in a meaningful way. All clinicians felt that only a small number of alerts were actionable, *“I would say maybe out of like ten people, maybe three were – need – required sort of action.”*

#### Alert Management Process

Clinicians’ responses to alerts varied widely because they had different criteria for responding. As noted in Fig. 1, some responded to every alert with a phone call, but only made clinical changes some of the time, typically based on symptomology. One challenge was the fact that alerts occurred whenever there were significant weight changes, rather than when there were trends in weight fluctuations, making them unreliable in identifying actual problems. Clinicians sometimes did not pay attention to alerts at “off” times or alerts that were lost in the shuffle of clinic flow, *“One is the weekend and two is that not every provider or every person who gets the alerts necessarily checks that area all the time.”* Additionally, some patients did not answer when the clinician’s office called, making clinical interventions difficult if not impossible. A couple of clinicians noted that the regularity of alerts could be overwhelming sometimes, *“I get a ton of inputs, I just can’t localize it to where I’m getting it from easily.”*

#### Patient Adherence

One quarter of clinicians noted that some patients may be more likely to benefit from a remote monitoring intervention than others because of their likelihood to be adherent; however, one respondent felt that the intervention helped patients manage their own health by giving them ownership of their wellbeing, *“get them used to taking ownership of their care.”* Twenty-five percent of clinicians thought that, while this intervention was helpful, patients must still be responsible for their health, *“I do sometimes have concerns with the patients then learning to take responsibility.”* A quarter of respondents also did not think that this, or similar interventions, would be helpful because patients do not understand their illness, and do not feel a sense of responsibility for their health. This was identified as a key barrier to success in the EMPOWER intervention, *“Most of the barriers that I see in my practice is adherence to medication and to diet and sodium restrictions.”*

## Discussion

The EMPOWER intervention did not reduce all-cause readmissions or mortality among HF patients who received the intervention, compared to control patients. Other studies have also found that - except for those that monitor pulmonary pressure - heart failure monitoring interventions have not been successful in improving health outcomes.^5–7^ However, despite the lack of a statistically significant effect of the intervention, patients appreciated it and felt it helped them.

The lottery also did not appear to impact the decision to enroll in the intervention and may not have had a strong enough effect on patient adherence to affect readmissions. Although patients said they did not choose to participate because of the lottery, many felt that it motivated them to pay attention to the weight and medication reminders. This suggests that the incentives for enrollment and for successful adherence once enrolled may be different.

Clinicians appreciated that the alerts fit well into the workflow of the EMR, but some found the number of alerts overwhelming and the way they were defined (short-term changes rather than trends) less helpful than they could be. Future work should explore more useful mechanisms for clinician engagement with alerts. Some clinicians felt that the alerts put too much responsibility on them and that, instead, patients needed to understand their condition better and take more responsibility. This view aligned with the responses of some patients, who said that they did not understand the connection between their weight, diuretic adherence, and their condition, suggesting that some patients need more education about managing their heart failure earlier during their condition.

Some clinicians responded to all alerts, while others responded only to those accompanied by symptoms, especially severe symptoms. Although the main trial paper found that alerts did predict readmission,^3^ no professional guidelines were, or are, available to tell clinicians how to respond to this new information. The variability in clinician responses may have impacted the results of the study as some patients may have benefited from more aggressive clinician intervention in response to alerts. More research to develop appropriate guidelines, training, and standardization around alert response protocols may reduce the variability in clinician response processes and improve patient outcomes.

### Limitations

We interviewed patients who were responsive to our outreach. The patients who participated in the intervention were more likely to be those who were highly adherent. Although we attempted to recruit additional low adherents, they were very difficult to reach. Similarly, clinicians we included in this study were those who agreed to participate in an interview and may not reflect the perspective of those who did not.

## Conclusion

Remote monitoring to reduce hospital readmissions has been implemented with varying degrees of success. In the EMPOWER study, an intervention to reduce hospital readmissions for patients with HF did not yield a statistically significant improvement.^3^ Our qualitative analysis indicates potential areas for additional exploration and consideration to design better interventions.

Many patients felt supported, and they appreciated the intervention. Many also appreciated the lottery intervention, and while it was not an incentive for enrolling for many respondents, it may have increased adherence during the study. The participants in this study were far along in their disease progression, and consideration of similar interventions to improve patient adherence earlier in disease progression may yield better results.

Clinicians felt that the intervention integrated well into their workflow, but the number of alerts was burdensome. Additionally, responses to alerts varied considerably by provider, perhaps because there are no professional guidelines for alerts unaccompanied by severe symptoms. Future work to identify useful responses to alerts by clinicians, and more clinically relevant criteria for triggering alerts, may help to achieve significant improvements in patient outcomes using remote monitoring techniques.

## Figures and Tables

**Table 1:** Recruitment

Interview Group	Recruitment Count	% Recruitment	Caucasian	% Caucasian	Black	Black	Other	% Other	Male	% Male	Female	% Female
High Adherence NO Readmission	20	32.26%	12	60.00%	7	35.00%	1	5.00%	7	35.00%	13	65.00%
High Adherence w/ Readmission	20	32.26%	14	70.00%	5	25.00%	1	5.00%	10	50.00%	10	50.00%
Low Adherence NO Readmission	12	19.35%	3	25.00%	9	75.00%	0	0.00%	6	50.00%	6	50.00%
Low Adherence w/ Readmission	10	16.13%	1	10.00%	9	90.00%	0	0.00%	5	50.00%	5	50.00%
Total	62	100.00%	30	48.39%	30	48.39%	2	3.23%	28	45.16%	34	54.84%

**Table 2:** Patient Respondents

Interview Group	Respondent Count	% Respondent	Caucasian	% Caucasian	Black	% Black	Other	% Other	Male	% Male	Female	% Female
High Adherence, NO Readmission	16	37.21%	9	56.25%	6	37.50%	1	6.25%	6	37.50%	10	62.50%
High Adherence WITH Readmission	17	39.53%	13	76.47%	3	17.65%	1	5.88%	9	52.94%	8	47.06%
Low Adherence, NO Readmission	7	16.28%	1	14.29%	6	85.71%	0	0.00%	4	57.14%	3	42.86%
Low Adherence WITH Readmission	3	6.98%	0	0.00%	3	100.00%	0	0.00%	1	33.33%	2	66.67%
TOTAL	43	100.00%	23	53.49%	18	41.86%	2	4.65%	20	46.51%	23	53.49%

**Table 3:** Patient Quote Table

**Health Perception**

*I had to always constantly go in the hospital... It just – it helped, the program. (LA/NR)*
*There’s been no shortness of breath or no swelling in my legs – because now I know what to look for when it comes to having congestive heart failure again and I haven’t had any of those symptoms at all for over a year now. (LA/NR)*
*Exercising more...more walking and less driving a lot times. A lot of times when I always go to the corner store, might be two or three blocks away, I used to drive. Then I decided to walk a lot more instead of driving to the corner store. Doing that will make me feel better and be healthier for me. (LA/WR)*
*Well, because I was weighing myself every day, I was knowledgeable of how much I weighed in the morning, so I knew how much I could eat during that day... (HA/ NR)*
*I would – I think I was still working a couple days a week at the time when I enrolled. So if it wasn’t a workday, I would get up in the morning and, in the beginning, I didn’t do too much because I couldn’t really walk up steps. I found it difficult to do that. But later on in the program, because I lost a lot of weight and I kept my weight down, I – my sodium level was very good, I had my energy back.... (HA/WR)*

Disease Management and Routine

*The pill bottle was the biggest asset in the program for me because I would forget to take my medication on a regular basis... the pill bottle every morning at 8 o’clock the buzzer would go off and light up purple and I knew it was time to take my medication. So that put structure in my life as far as taking my medication. (LA/NR)*
*The program really kept me on the ball as far as taking my medicine, and plus they checking my weight... it made me look forward to it every day. (LA/WR)*
*... I still weigh myself everyday still. I guess I got into the habit, so it’s kinda hard to break. The pill bottle, I did throw in the trash because the pill bottle was like, it was kinda – it was a little heavy. So, I kinda, I threw that in the trash, but yeah. (H/NR)*
*I thought it was great, it was very helpful. I mean, it actually reminded me to take the pills. And I would step on the scale, I was more conscious of stepping on the scale.... (HA/WR)*

Effectiveness

*I think it kinda helped me stay out of the hospital because... it would send the messages to my doctor when I gained the weight and they would call me and tell me like take an extra pill, take a half a pill. Whatever to do to try to get the extra water weight...(HA/NR)*
*I think it prevented me, probably, maybe from lapsing into the bad health. I was in the hospital when I got put on the program. It kept me from going back into that state that I was in, which was a bad state brought on by bad eating, lack of moving, lack of exercise...the program, which got me to maintaining, stabilizing a weight instead of going up or going down on a steady basis down, steady basis up too high. It helped keep me in line, eating the way I should so that I would stay at the weight I was supposed to stay for optimum health with the heart. (HA/R)*
*It kept me out of the hospital, it kept me a little bit more healthier and it always reminded me when I forgot. (LA/NR)*

Lottery

*It didn’t change how I felt about the program. I was still going to be in it. But it just made it a little bit better... (LA/WR)*
*... I mean, when you have a condition like mine – when you damn near die, you get kind of scared and you just do what you’re supposed to do to try to take care of yourself. So it was more a case of making sure I did what I was supposed to do as opposed to worrying about whether I was going to get money for it. (HA/WR)*
*I did get some days that... today I’ve made this much money or whatever. And it was kind of like, yes, I took my medicine yesterday and stepped on the scale. And then it’s like – so it was kind of like a reward for doing that, that way, so it was a nice little thing to see, and it was like, yes, I did what I was supposed to do yesterday and it’s like – and got rewarded for doing so. (HA/WR)*
*Well, I mean, it was fun. The little monies here and there, the little checks here and there with the lottery was ...the little money was on top, but it wasn’t never about the money. It was always about – but I think initially it helped motivate me, those little couple of dollars. But eventually I don’t even think it was about the money, it was about me learning how to adapt to my sickness. (HA/NR)*
